# Biexponential *I* = 3/2 Spin–Lattice Relaxation in the Solid State: Multiple-Quantum ^7^Li NMR as a Probe of Fast Ion Dynamics

**DOI:** 10.1021/acs.jpcc.4c00262

**Published:** 2024-03-26

**Authors:** Stephen Wimperis, George E. Rudman, Karen E. Johnston

**Affiliations:** † Department of Chemistry, Faraday Building, Lancaster University, Lancaster LA1 4YB, U.K.; ‡ Department of Chemistry, Durham University, Durham DH1 3LE, U.K.

## Abstract

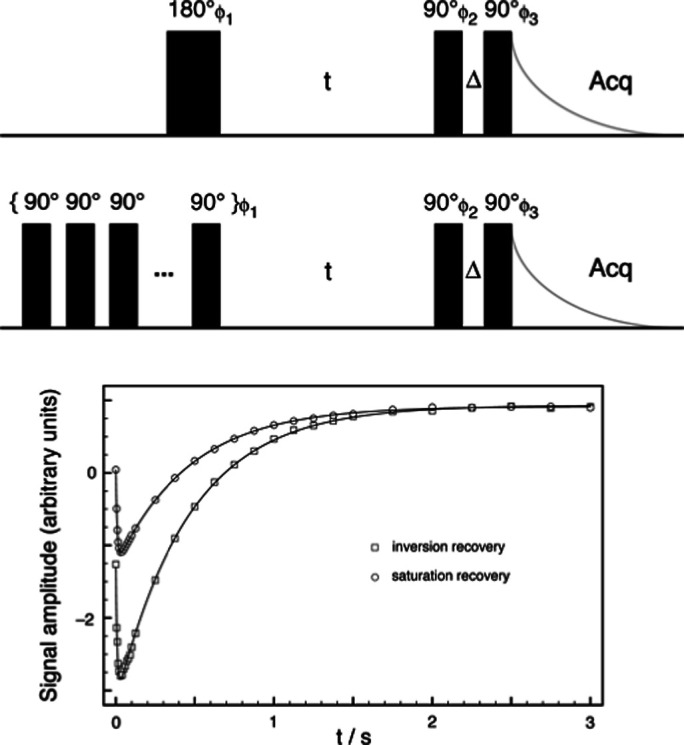

Spin–lattice relaxation measurements are used in ^7^Li NMR studies of materials of potential use in solid-state Li-ion batteries as a probe of ion mobility on a fast (nanosecond to picosecond) time scale. The relaxation behavior is often analyzed by assuming exponential behavior or, equivalently, a single *T*_1_ time constant. However, the spin–lattice relaxation of spin *I* = 3/2 nuclei, such as ^7^Li, is in general biexponential; this is a fundamental property of *I* = 3/2 nuclei and unrelated to any compartmentalization within the solid. Although the possibility of biexponential ^7^Li (and other *I* = 3/2 nuclei) spin–lattice relaxation in the solid state has been noted by a number of authors, it can be difficult to observe unambiguously using conventional experimental NMR techniques, such as inversion or saturation recovery. In this work, we show that triple-quantum-filtered NMR experiments, as previously exploited in *I* = 3/2 NMR of liquids, can be used in favorable circumstances to observe and readily quantify biexponential ^7^Li spin–lattice relaxation in solids with high ion mobility. We demonstrate a triple-quantum-filtered inversion-recovery experiment on the candidate solid electrolyte material Li_2_OHCl at 325 K, which has previously been shown to exhibit fast ion mobility, and we also introduce a novel triple-quantum-filtered saturation-recovery experiment. The results of these solid-state NMR experiments are less straightforward than those in liquids as a consequence of the unwanted direct excitation of triple-quantum coherences by the weak (compared with the unaveraged ^7^Li quadrupolar interaction) pulses used, but we show that this unwanted excitation can be accounted for and, in the example shown here, does not impede the extraction of the two ^7^Li spin–lattice relaxation times.

## 1 Introduction

The NMR relaxation of quadrupolar nuclei with spin quantum number *I* ≥ 3/2 is, in general, multiexponential.^1,2^ The phenomenon has been extensively investigated and discussed, in particular in the context of ^7^Li, ^23^Na, ^39^K, and ^87^Rb (spin *I* = 3/2) and ^25^Mg and ^17^O (spin *I* = 5/2) NMR studies of the binding of simple cations and water to macromolecules in solution.^3−9^ The deviation from monoexponential behavior is often significant and easily observable for spin–spin relaxation.^3−5^ However, in the case of the more commonly studied spin–lattice relaxation, the deviation is usually smaller and can be difficult to detect unless multiple-quantum filtration techniques are used.^5−7,9^

In NMR of solids, monoexponential spin–lattice relaxation of quadrupolar nuclei has often been assumed. In his major review from 1978, Spiess, following Abragam, noted that the relaxation of spin *I* ≥ 3/2 nuclei should be nonexponential, i.e., multiexponential, described by multiple relaxation rate constants.^1,10^ However, he then explicitly chose to disregard this possibility “for the sake of the argument” (ref (10)., p 113) and present theoretical expressions for single relaxation rate constants for both spin–spin and spin–lattice quadrupolar relaxation.^10^ These expressions are correct for spin *I* = 1 nuclei and for spin *I* ≥ 3/2 nuclei in the fast-motion or extreme-narrowing limit, where the relevant motional correlation time τ_c_ is much smaller than the inverse of the Larmor frequency, ω_0_^–1^. However, outside the extreme-narrowing limit, when the motion is slower and the condition ω_0_τ_c_ ≪ 1 does not hold, the Spiess expressions with their single rate or time constants can, at best, be only approximately correct for spin *I* ≥ 3/2 nuclei.^1,10^

In recent years, there has been much interest in using solid-state ^7^Li (spin *I* = 3/2) NMR to investigate the structure and properties of candidate materials for Li-ion batteries.^11−16^ Studies of the mobility of lithium ions over a range of time scales in solid materials yield an increased understanding of ion dynamics that should eventually aid in the development of solid-state ion conductors with performance superior to those available currently. In particular, fast lithium-ion dynamics (on the nanosecond or picosecond time scale) are often studied by means of ^7^Li NMR spin–lattice relaxation measurements.^11,13,15−17^ Although a number of authors have pointed out the possibility of biexponential ^7^Li (and other *I* = 3/2 nuclei) spin–lattice relaxation,^13,18^ in general, monoexponential relaxation has usually been assumed, with use of the Spiess equation being prominent.

In this work, we demonstrate that, in favorable circumstances, biexponential ^7^Li spin–lattice relaxation can be observed and quantified in the solid state using the same triple-quantum-filtered inversion-recovery experiment that has been employed for *I* = 3/2 NMR of solutions and other liquids.^5,6,9^ Furthermore, it will be shown that a triple-quantum-filtered saturation-recovery experiment can also be used, offering considerable time saving. However, when interpreting the results of these experiments, we will show that allowance has to be made for the finite strength of the radiofrequency pulses used when compared to the unaveraged ^7^Li quadrupolar interaction. As a suitable model system for developing these NMR methods, we have chosen to study Li_2_OHCl at 325 K, as this previously investigated cubic phase is known to exhibit fast ion mobility (∼10^–4^ S cm^–1^).^14,15,19^ It should be emphasized that the biexponential spin–lattice relaxation observed here is an inherent property of the spin *I* = 3/2 nucleus and is not associated with there being two distinct pools or compartments of monoexponentially relaxing ^7^Li nuclei within the solid.

## 2 Theory

### 2.1 Biexponential *I* = 3/2 Spin–Lattice Relaxation

According to Spiess (ref (10)., Table 4.4, p 117), the quadrupolar spin–lattice relaxation rate constant for a spin *I* nucleus is given by^10^
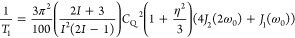
1where the quadrupolar coupling constant (in Hz) is
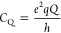
2The spectral density functions *J*_1_(ω) and *J*_2_(ω) are the Fourier transforms of the relevant autocorrelation functions for the fluctuating quadrupolar interaction.^20^ The two functions are in general different, corresponding to different components of the quadrupolar interaction tensor, but for the simplest case of isotropic modulation of the quadrupolar interaction with an exponential correlation function, we can write
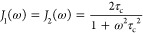
3where τ_c_ is the correlation time. For the particular case of *I* = 3/2, eq 1 reduces to
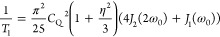
4Note, however, that this is at best only an approximate expression, as the possibility of multiexponential relaxation has been excluded.

Following the approach of Jaccard et al., it is possible to present an exact expression for *I* = 3/2 quadrupolar spin–lattice relaxation.^5^ Using spherical tensor operators,^21^ we can write the *I* = 3/2 thermal equilibrium density operator as
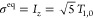
5and the perturbed initial state at the start of a spin–lattice relaxation experiment as
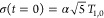
6where α = −1 for inversion recovery and α = 0 for saturation recovery. The deviation from thermal equilibrium can then be written as
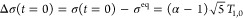
7According to Jaccard et al.,^5^ the equation of motion for this deviation is
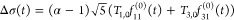
8where
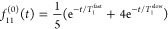
9a
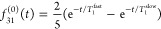
9bThese biexponential functions contain two quadrupolar spin–lattice relaxation rate constants
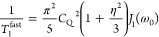
10a
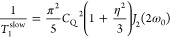
10b

Following inversion or saturation, the relaxation function  can be measured using a 63.4° readout pulse to null the contribution from the *T*_3,0_ component.^22^ Note that the function  may appear to be similar to a single exponential: the two rate constants are often similar, while the component with the rate constant  occurs with four times the amplitude of the other component. In the initial rate approximation, where *t* ≈ 0,  reduces to an approximate single exponential with the rate constant given in eq 4.

### 2.2 Measurement of *I* = 3/2 Biexponential Relaxation in Liquids

As shown by Jaccard et al.,^5^ the key to the unambiguous observation and measurement of *I* = 3/2 biexponential quadrupolar spin–lattice relaxation lies in the *T*_3,0_ tensor component of Δσ(*t*) in eq 8. This represents an octupolar spin population distribution.^5^ Unusually, it does not correspond to a spin temperature.^1^ It can only arise when the function  is nonzero and this only occurs when , as the two exponentials in this function have the same magnitude at *t* = 0 but opposite signs.

**Figure 1:**
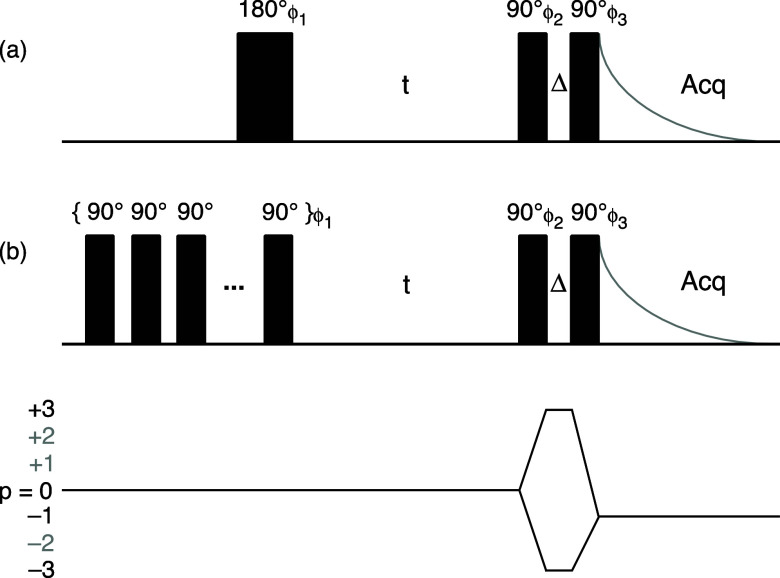
Pulse sequences and coherence transfer pathway diagram for triple-quantum filtration of spin *I* = 3/2 nuclei undergoing spin–lattice relaxation. The sequence in (a) is for an inversion-recovery experiment and that in (b) for a saturation-recovery experiment (commencing with a train of, typically, twenty 90° pulses). A 24-step phase cycle was used in each experiment: ϕ_1_ = 24 × 0°, ϕ_2_ = 30, 90, 150, 210, 270, 330, 120, 180, 240, 300, 0, 60, 210, 270, 330, 30, 90, 150, 300, 0, 60, 120, 180, and 240°, and ϕ_3_ = 6 × 0°, 6 × 90°, 6 × 180°, and 6 × 270° with the receiver phase ϕ_Rx_ = 3 × {0°, 180°}, 3 × {90°, 270°}, 3 × {180°, 0°}, and 3 × {270°, 90°}. Note that ϕ_2_ and ϕ_3_ are always offset from each other by an odd multiple of 30° to ensure that the signals originating from the *p* = +3 and −3 coherences do not cancel each other.^6^ Biexponential spin–lattice relaxation is studied as a function of the variable recovery period *t*. The fixed period Δ is typically just 2 μs to allow for phase shifting of the pulses.

An intense radiofrequency pulse (defined as one where the radiofrequency field strength ω_1_ is many orders of magnitude greater than any resolved quadrupolar splitting parameter, ω_Q_, so ω_1_ ≫ ω_Q_) can change the coherence order p of a tensor operator *T*_l,p_ but not its spin rank l.^5,21^ Therefore, an intense 90° pulse applied to a state described by a tensor operator *T*_3,0_ will create triple-quantum coherence *T*_3,±3_ (and other coherences). This triple-quantum coherence can be separated from other coherences and population states by phase cycling and then reconverted into observable *p* = −1 coherence, in this case, *T*_3,–1_, by a second 90° pulse. No triple-quantum coherences can be excited from *T*_1,0_ by an intense NMR pulse and so by using this two-pulse triple-quantum filter after an inversion pulse or saturation train, as shown in the pulse sequences in Figure 1, the relaxation function , describing the build up and decay of the octupolar *T*_3,0_ state, can be measured as a function of the period *t* between the saturation or inversion event and the triple-quantum filter.^5,6^ The density operator immediately after the final pulse can be written as
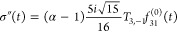
11and is only nonzero when , i.e., when biexponential spin–lattice relaxation has occurred.^5,6^

### 2.3 Measurement of *I* = 3/2 Biexponential Relaxation in Solids

Attempting to use triple-quantum filtration to observe and measure *I* = 3/2 biexponential spin–lattice relaxation in solids raises questions and presents some difficulties. First, there is the question of whether biexponential quadrupolar spin–lattice relaxation occurs in solids. It is possible that dipole–dipole interactions between the quadrupolar nuclei might lead to rapid spin diffusion and hence maintenance of a uniform spin temperature,^1^ preventing any buildup of the octupolar T_3,0_ population distribution. This seems unlikely, however, in the context of ^7^Li NMR studies of lithium-ion dynamics in solids where high ion mobility is suspected; rapid motion of the ^7^Li nuclei would average the homonuclear dipole–dipole interactions and so prevent efficient spin diffusion, as occurs in liquids. Indeed, the ^7^Li NMR spectra of solids where high ion mobility is found often resemble those of liquids.

A more practical difficulty is that the radiofrequency pulses used in NMR of quadrupolar nuclei in solids are not intense, i.e., the resolved quadrupolar splitting parameter ω_Q_ is usually large enough that the condition ω_1_ ≫ ω_Q_ does not hold. In fact, for many quadrupolar nuclei, such as ^17^O, ^23^Na, or ^27^Al, the condition ω_Q_ ≫ ω_1_ is more likely to apply. In this case, an NMR pulse will be able to change the spin rank l of a tensor operator *T*_l,p_ as well as its coherence order p. For example, a nonintense radiofrequency pulse applied to an equilibrium density operator proportional to *T*_1,0_ can excite triple-quantum coherence *T*_3,±3_ directly,^23,24^ a phenomenon widely exploited in the well-known multiple-quantum MAS (MQMAS) experiment.^25−27^ This means that any triple-quantum-filtered signal observed in an experiment such as those in Figure 1 may arise from triple-quantum coherences excited directly by the first pulse in the triple-quantum filter from the *T*_1,0_ component as well as from those excited directly from the *T*_3,0_ component, as intended. (Note that any triple-quantum coherences inadvertently excited by an imperfect inversion pulse or saturation train can be removed by phase cycling^28^ to select *p* = 0 during the recovery interval *t* as shown in Figure 1, while any excited by the final pulse will not be observable in the free induction decay.)

The effect of a nonintense first filter pulse acting on a biexponentially relaxing *I* = 3/2 spin system can be modeled analytically. Using eqs 5 and 8, we can write the density operator after the initial perturbation and relaxation as
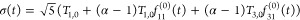
12The Hamiltonian during the nonintense pulse is
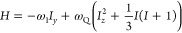
13where we have chosen the pulse phase to be −*y* arbitrarily (so the result of our calculation will be real rather than imaginary). The effect of such a pulse on the density operator in eq 12 can then be written as
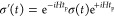
14where *t*_p_ is the pulse duration. In ^7^Li NMR studies of a solid with high ion mobility, we would expect ω_Q_, although nonzero, to be smaller than ω_1_ and so the optimum pulse duration in the triple-quantum filter would be close to that of an intense 90° pulse, such that ω_1_*t*_p_ = π/2. Hence, we can set
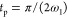
15and compute σ′ (t) in eq 14 analytically in matrix form (with the aid of Maple in our case^29^) to obtain a result containing only the dimensionless variables α, , , and the ratio ω_Q_/ω_1_. We are interested in the amplitude of triple-quantum coherence excited by the pulse, and this is represented by the (equal) amplitudes of the density matrix elements . We can thus extract the density matrix element  and use it as a measure of the excitation of triple-quantum coherence. Unfortunately, this element, although useable for calculations, is too lengthy to give here in full analytical form; instead, we present it in full in the Supporting Information (S1).

For ^7^Li NMR studies of a solid with high ion mobility, we would expect ω_Q_/ω_1_ < 1 (and we have already assumed this in our choice of the pulse duration) and so, for the purposes of illustration, can make a Maclaurin series expansion of  to second order in ω_Q_/ω_1_, yielding
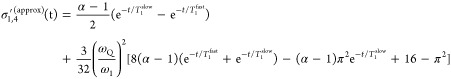
16The next term in the series is on the order of (ω_Q_/ω_1_)^4^, indicating likely strong convergence. If ω_Q_ = 0, we can see that only the first term in the series is nonzero, and so this represents the amplitude of triple-quantum coherence that would be excited by an intense 90° radiofrequency pulse, as usually found in the NMR of liquids. In other words, this first term represents the amplitude of triple-quantum coherence excited purely from the *T*_3,0_ component of the density operator and hence having a time dependence proportional to the function . By contrast, the second term in the series, proportional to (ω_Q_/ω_1_)^2^, represents the additional (and unwanted) amplitude of triple-quantum coherence excited by a nonintense pulse from both the *T*_1,0_ and *T*_3,0_ components present in the relaxing density operator. As expected, this second term includes both time-dependent and time-independent components, with the latter arising from the thermal equilibrium amount of operator *T*_1,0_ remaining at long *t* values. In the Supporting Information (S2), we show that the spin–lattice relaxation curves predicted by eq 16 are in excellent agreement with the exact curves for ω_Q_/ω_1_ < 0.5.

**Figure 2:**
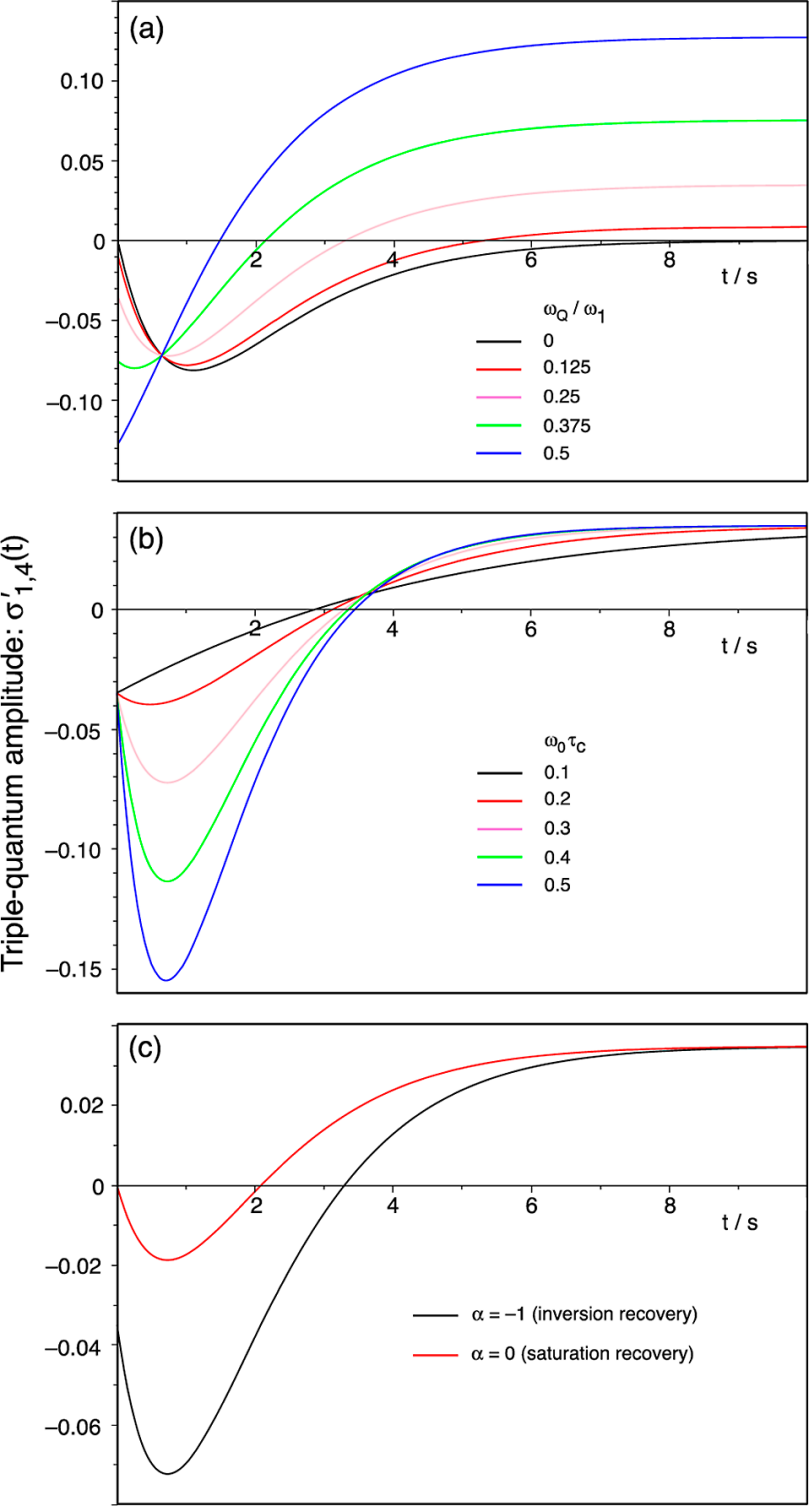
Plots of  from eq S1 in the Supporting Information (a theoretical measure of the amplitude of *I* = 3/2 triple-quantum coherence excited by a 90° pulse) as a function of the spin–lattice relaxation interval *t* in a triple-quantum-filtered relaxation experiment. Results are shown for a variety of conditions that might be typical of a ^7^Li NMR experiment performed on a material with high ion mobility, including *C*_Q_ = 30 kHz, η = 0, and ω_0_/(2π) = 155 MHz. In (a),  is plotted as a function of *t* for α = −1 (i.e., an inversion-recovery experiment) and ω_0_τ_c_ = 0.3 for a range of ratios ω_Q_/ω_1_ from 0 to 0.5 (with 0 representing an intense radiofrequency pulse, ω_1_ ≫ ω_Q_). In (b),  is plotted as a function of *t* for α = −1 and ω_Q_/ω_1_ = 0.25 for a range of motional correlation parameters ω_0_τ_c_ from 0.1 to 0.5 (with 0.1 representing faster and 0.5 slower motion). And in (c),  is plotted as a function of *t* for ω_Q_/ω_1_ = 0.25 and ω_0_τ_c_ = 0.3 for both α = −1 (i.e., an inversion-recovery experiment) and α = 0 (i.e., a saturation-recovery experiment).

Plots of  from (exact) eq S1 in the Supporting Information (S1) as a function of the relaxation interval *t* are shown in Figure 2 for a variety of conditions that might be typical of a ^7^Li NMR experiment performed on a material with high ion mobility. We have assumed isotropic modulation of the quadrupolar interaction with an exponentially decaying correlation function, as in eq 3. In Figure 2a,  is plotted as a function of *t* for α = −1 (i.e., an inversion-recovery experiment), *C*_Q_ = 30 kHz, quadrupolar asymmetry parameter η = 0, ω_0_/(2π) = 155 MHz, and ω_0_τ_c_ = 0.3 and for a range of ratios ω_Q_/ω_1_ from 0 to 0.5. In Figure 2b,  is plotted as a function of *t* for α = −1, *C*_Q_ = 30 kHz, η = 0, ω_0_/(2π) = 155 MHz, and ω_Q_/ω_1_ = 0.25 for a range of motional correlation parameters ω_0_τ_c_ from 0.1 to 0.5. And in Figure 2c,  is plotted as a function of *t* for *C*_Q_ = 30 kHz, η = 0, ω_0_/(2π) = 155 MHz, ω_Q_/ω_1_ = 0.25, and ω_0_τ_c_ = 0.3 for both α = −1 (i.e., an inversion-recovery experiment) and α = 0 (i.e., a saturation-recovery experiment). These theoretical models will be compared with the experimental NMR results in the Results and Discussion section.

## 3 Materials and Methods

Li_2_OHCl was synthesized via solid-state methods. Stoichiometric amounts of commercial LiCl (Alfa Aesar, ultradry, 99.9%) and LiOH (Acros Organics, anhydrous, 98%) were mixed and ground in an agate mortar and pestle inside an argon-filled glovebox. The powder was placed in an alumina crucible and heated at 350 °C for 30 min in a muffle furnace located inside an argon-filled glovebox. Once the reaction was complete, the furnace was allowed to cool to room temperature and the material was recovered and characterized by powder X-ray diffraction (PXRD) using a Bruker D8 diffractometer using Mo (λ = 0.71073 Å) radiation. The characterization and structural data for the prepared sample are given in the Supporting Information (S3).

A sample of Li_2_OHCl was packed in a Bruker 4-mm MAS NMR rotor under argon. Solid-state NMR experiments were performed on a Bruker 400 AVANCE III HD spectrometer equipped with a wide-bore 9.4 T magnet at a Larmor frequency of ω_0_/(2π) = 155.5 MHz for ^7^Li. The MAS rate was either 12 kHz or 0 (for static experiments). Proton decoupling was observed to make only a negligible difference to the ^7^Li NMR line width and so was not used. The sample temperature was 325 K, with allowance being made for sample heating under MAS. At this temperature, Li_2_OHCl exists in a cubic antiperovskite phase with space group *Pm*3®*m* where high Li-ion mobility has been observed.^14^ The chemical shift scale was referenced to 1 M LiCl_(aq)_ at 0 ppm. The 180° pulse duration calibrated on Li_2_OHCl was 4.5 μs, implying a radiofrequency field strength of ω_1_/(2π) = 110 kHz for all pulses used in our experiments. A 24-step phase cycle was used for the triple-quantum filtration experiments: a 6-step cycle to select *p* = ± 3 coherences between the two filter pulses was nested with a 4-step cycle to ensure *p* = 0 populations during the relaxation interval between the inversion or saturation recovery and the triple-quantum filter. Note that the phases of the two 90° filter pulses must be offset from each other by an odd multiple of 30° to ensure that the signals originating from the *p* = +3 and −3 coherences do not cancel each other.^6^ After a preliminary investigation of the sample, a recycle interval of 3 s was used for the conventional pulse-acquire and triple-quantum-filtered inversion-recovery experiments, while 0.5 s was used for the saturation-recovery experiments.

## 4 Results and Discussion

**Figure 3:**
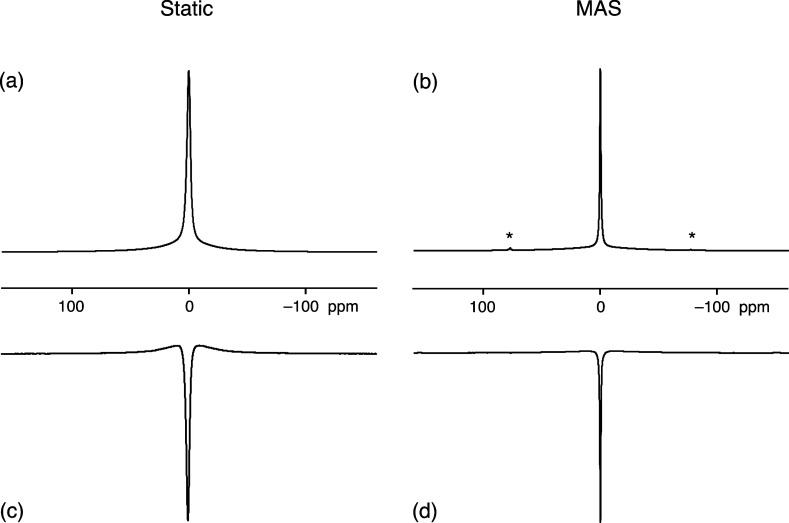
^7^Li NMR spectra of Li_2_OHCl recorded at 325 K and a Larmor frequency of 155.5 MHz. The spectral width shown is 50 kHz (322 ppm). (a, b) Conventional pulse-acquire spectra, recorded (a) under static conditions and (b) using MAS at 12 kHz. Small spinning sidebands are marked with * in (b). Number of transients coadded: 16. (c, d) Spectra recorded using the triple-quantum-filtered inversion-recovery experiment in Figure 1a with *t* = 40 ms; (c) under static conditions and (d) using MAS at 12 kHz. Number of transients coadded: 504.

Figure 3a,b shows conventional ^7^Li static and MAS NMR spectra of Li_2_OHCl recorded at 325 K and is in agreement with previous studies.^14^ The spectra are similar to many *I* = 3/2 NMR spectra in liquids, exhibiting a single resonance but with two components, one broad and one narrow. In solution-state NMR, these two components would be assumed to be homogeneous and attributed to biexponential spin–spin relaxation. However, in solids, one must be more cautious. For example, the narrow component narrows further under MAS, indicating an inhomogeneous contribution to the line width, while small spinning sidebands are also visible in Figure 3b.

Triple-quantum-filtered ^7^Li static and MAS NMR spectra of Li_2_OHCl recorded with the inversion-recovery sequence in Figure 1a using a recovery interval of *t* = 40 ms are shown in Figure 3c,d. These spectra show the expected line shape for a triple-quantum-filtered *I* = 3/2 NMR spectrum,^5,6^ with the narrow component now having the opposite phase to the broad component.

**Figure 4:**
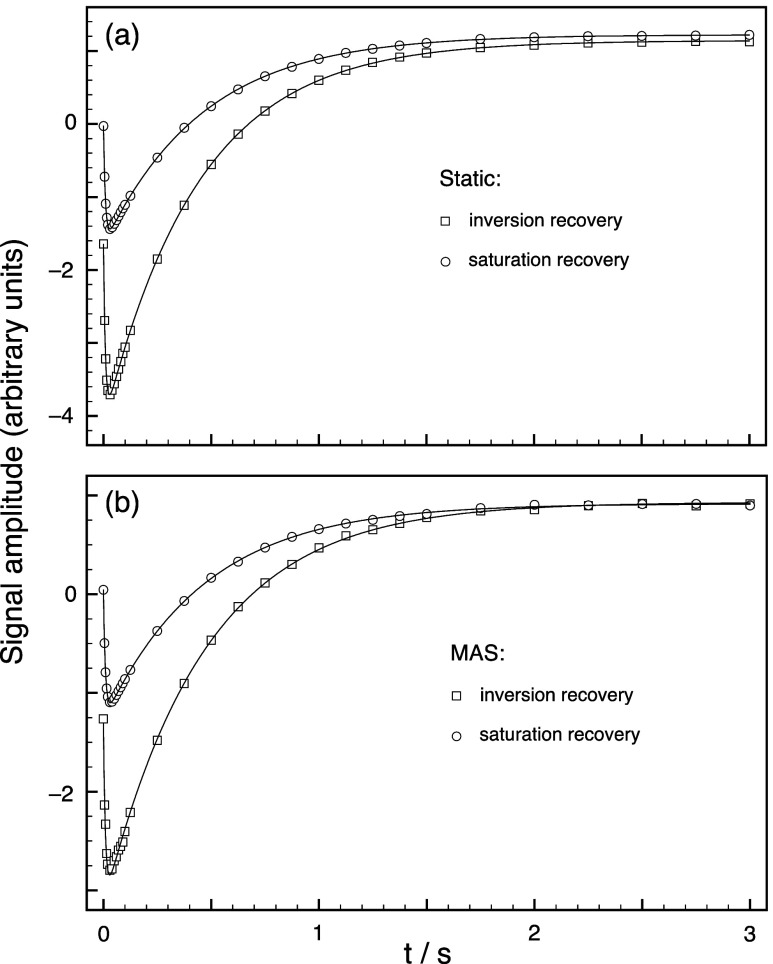
^7^Li NMR signal amplitudes (squares for inversion recovery and circles for saturation recovery) as a function of the recovery interval *t* in the triple-quantum-filtered relaxation experiments in Figure 1. The sample was Li_2_OHCl and spectra were recorded at a temperature of 325 K and a Larmor frequency of 155.5 MHz; (a) under static conditions and (b) using MAS at 12 kHz. Number of transients coadded for each data point: 504. The solid lines are fittings of the experimental data points with a biexponential function plus a constant as described in the text.

Figure 4 shows plots of the ^7^Li NMR signal amplitude (the amplitude of the narrow component of the ^7^Li line shape) from Li_2_OHCl as a function of the recovery interval *t* in the triple-quantum-filtered relaxation experiments in Figure 1. The static results in Figure 4a are essentially identical with the MAS results in Figure 4b. This is as one would expect for a spin–lattice relaxation process driven by fluctuations of the quadrupolar interaction with frequencies similar to the Larmor frequency of 155.5 MHz and so unaffected by a modulation of the quadrupolar interaction with the MAS frequency of 12 kHz.

The experimental data in Figure 4 can be compared with the theoretical curves in Figure 2 and the following points can be noted.

(i) The triple-quantum-filtered inversion-recovery experiment (square data points in Figure 4) yields signal at *t* = 0. This is in contrast to what would be expected in liquids (the ω_Q_/ω_1_ = 0 curve in Figure 2a) where there is no signal at *t* = 0 because spin–lattice relaxation has not had the time to produce any *T*_3,0_ component of the density operator. Hence, the signal at *t* = 0 in the inversion-recovery data in Figure 4 is unwanted and arises from triple-quantum coherences excited directly from the (inverted) *T*_1,0_ component of the density operator by the finite-intensity first pulse in the triple-quantum filter.
(ii) Similarly, all the experimental data in Figure 4 show signals at full relaxation (e.g., at *t* = 3 s), corresponding to unwanted direct excitation of triple-quantum coherences from (thermal equilibrium) *T*_1,0_, whereas the ω_Q_/ω_1_ = 0 curve in Figure 2a decays to zero at long *t* values as the *T*_3,0_ component of the density operator relaxes away.
(iii) The triple-quantum-filtered saturation-recovery experiment (circle data points in Figure 4) yields no signal at *t* = 0 because, as expected, the saturation train of pulses has destroyed all ^7^Li magnetization in the sample.
(iv) The shape of the triple-quantum-filtered recovery curves in Figure 4, both for inversion recovery and saturation recovery, can be compared to those in Figure 2c and seen to have a similar form. These curves can be understood as the sum of triple-quantum-filtered signal arising purely from *T*_3,0_ produced by biexponential spin–lattice relaxation (the ω_Q_/ω_1_ = 0 curve in Figure 2a or, equivalently, the first term in eq 16) and triple-quantum-filtered signal arising from unwanted excitation from (chiefly) the *T*_1,0_ component of the density operator. Crucially, the former component shows a minimum at *t* > 0 in its recovery curve, while the latter shows no such minimum.
(v) The presence of a *t* > 0 minimum in the triple-quantum-filtered recovery curve in the results in Figure 4 is diagnostic of biexponential ^7^Li spin–lattice relaxation. However, even when present, such relaxation may not always produce a minimum. For example, in Figure 2a, it can be seen that the recovery curve for ω_Q_/ω_1_ = 0.5 shows no minimum because the unwanted excitation of triple-quantum coherence directly from *T*_1,0_ dominates the excitation from *T*_3,0_ for this relatively weak pulse. Similarly, in Figure 2b, it can be seen that the recovery curve for ω_0_τ_c_ = 0.1 shows no minimum because the two exponential components are very similar under these very fast motional conditions and there is little excitation of triple-quantum coherences from the only small amount of *T*_3,0_ in this case but still a significant amount of unwanted excitation from *T*_1,0_.

Theoretically, we would expect all the recovery curves in Figures 2 and 4 to be of the form
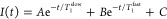
17(see eqs 16 and S1 in the Supporting Information), where *I*(*t*) is the signal amplitude, *A* and *B* are the amplitudes of the two exponential components, and *C* is a constant corresponding to the (unwanted) signal amplitude at long *t* values arising from excitation of triple-quantum coherences from equilibrium *T*_1,0_. The four sets of experimental data in Figure 4 have been fitted with this function (using pro Fit in our case^30^), as shown by the solid curves, yielding the spin–lattice relaxation time constants  = 441–468 ms and  = 8.65–9.91 ms. The difference between these time constants is much larger than the maximum factor of 4 predicted for ω_0_τ_c_ ≫ 1 under the assumption of isotropic modulation of the quadrupolar interaction with an exponentially decaying correlation function (see eq 3). Therefore, unsurprisingly, in view of the properties of Li_2_OHCl as a solid-state ionic conductor, where there must be translational diffusion of the lithium ions,^14^ it seems that such a simplistic rotational-only model of ionic motion is not applicable in this case. We envisage further work to interpret measurements of biexponential ^7^Li spin–lattice relaxation times in Li_2_OHCl and similar materials using more realistic motional models.

## 5 Conclusions

We have shown, using ^7^Li NMR of Li_2_OHCl as an example, that biexponential *I* = 3/2 spin–lattice relaxation can occur in solids, i.e., it is not necessarily swamped by homonuclear spin-diffusion, and that, under favorable experimental circumstances, it can be unambiguously detected using the same triple-quantum-filtered techniques, inversion recovery and saturation recovery, that can be employed in liquid or solution-state NMR.

In liquids, detection of any NMR signal in a triple-quantum-filtered inversion-recovery or saturation-recovery experiment on a *I* = 3/2 nucleus would indicate the presence of the *T*_3,0_ operator and, hence, of biexponential spin–lattice relaxation. However, this is not the case in solids, where the finite power of the radiofrequency pulses used relative to the magnitude of the unaveraged quadrupolar interaction means that there is always some unwanted excitation of triple-quantum coherences from the *T*_1,0_ component of the density operator. Instead, in solids, the observation of a minimum (or maximum, depending on spectrum phasing) turning point in the recovery curve can be taken as indicative of biexponential spin–lattice relaxation.

However, for this turning point in the triple-quantum-filtered relaxation curve to be observed, we have demonstrated that the radiofrequency pulses must be fairly strong compared with the quadrupolar interaction. It would appear that these conditions can often be met in solid-state ^7^Li NMR of high Li-ion mobility materials as the ^7^Li gyromagnetic ratio is large (hence the pulses can be fairly strong) while the ^7^Li quadrupolar moment is relatively small (hence the ^7^Li quadrupolar interaction is small and will be further averaged by the ion dynamics).

Biexponential ^7^Li spin–lattice relaxation may, of course, be observable in carefully performed and analyzed conventional inversion-recovery and saturation-recovery experiments and with much higher NMR sensitivity than using the triple-quantum-filtered experiments demonstrated here. However, the origin of this biexponential behavior would remain uncertain in such an experiment: for example, it could be due to the presence of two distinct compartments of Li ions in the solid, both exhibiting monoexponential relaxation in the extreme-narrowing limit, ω_0_τ_c_ ≪ 1. Furthermore, a conventional spin–lattice relaxation experiment does not produce the turning point in the recovery curve that we have demonstrated here in Figure 4, indicative of the two exponential components having opposite signs rather than the same sign. The presence of this turning point, in addition to being diagnostic of the presence of *I* = 3/2 biexponential spin–lattice relaxation, also makes any fitting of the curve to a biexponential function inherently more reliable.

## Supporting Information Available

Full form of the *I* = 3/2 triple-quantum density matrix element ; comparison of spin–lattice relaxation curves calculated using the exact expression in eq S1 and the approximate expression in eq 16; and crystallographic and powder X-ray data for the prepared sample of Li_2_OHCl (PDF)
