# Supplementary material for: Biexponential I = 3/2 Spin–Lattice Relaxation in the Solid State: Multiple-Quantum 7Li NMR as a Probe of Fast Ion Dynamics
Source: J Phys Chem C Nanomater Interfaces. 2024 Mar 26;128(13):5453–60. doi: 10.1021/acs.jpcc.4c00262 (PMC11000215; doi:10.1021/acs.jpcc.4c00262)
Supplement: Supplementary file 1 — jp4c00262_si_001.pdf [file jp4c00262_si_001.pdf]

Biexponential I = 3/2 Spin-Lattice Relaxation in the Solid State: Multiple-Quantum ^{7}Li NMR as a Probe of Fast Ion Dynamics

Stephen Wimperis,[*,a] George E. Rudman,[b] Karen E. Johnston[b]

[a]*Department of Chemistry, Faraday Building, Lancaster University,*

Lancaster LA1 4YB, United Kingdom

[b]*Department of Chemistry, Durham University,*

Durham, DH1 3LE, United Kingdom

* Corresponding author. Email: *s.wimperis@lancaster.ac.uk*

orcid.org/0000-0003-3009-5146

Supporting Information

S1. **Full form of the I = 3/2 triple-quantum density matrix element $\sigma'_{1,4}(t)$**

S2. **Comparison of spin-lattice relaxation curves calculated using the exact expression in eq S1 and the approximate expression in eq 16**

S3. **Crystallographic and powder X-ray data for the prepared sample of Li_2OHCl**

S1. Full form of the I = 3/2 triple-quantum density matrix element $\sigma'_{1,4}(t)$

$$\sigma'_{1,4}(t)=\frac{AB\left(3+(\alpha-1)e^{-\frac{t}{T_1^{fast}}}+2(\alpha-1)e^{-\frac{t}{T_1^{slow}}}\right)\cos\left(\frac{\pi A}{2}\right)\cos\left(\frac{\pi B}{2}\right)\;+\;\left(3\left(\frac{\omega_Q}{\omega_1}\right)^2+(\alpha-1)\left(\left(\frac{\omega_Q}{\omega_1}\right)^2-1\right)e^{-\frac{t}{T_1^{fast}}}+(\alpha-1)\left(2\left(\frac{\omega_Q}{\omega_1}\right)^2+1\right)e^{-\frac{t}{T_1^{slow}}}\right)\sin\left(\frac{\pi A}{2}\right)\sin\left(\frac{\pi B}{2}\right)}{2AB} \tag{S1a}$$

with

$$A=\sqrt{\left(\frac{\omega_Q}{\omega_1}\right)^2+\frac{\omega_Q}{\omega_1}+1} \tag{S1b}$$

and

$$B=\sqrt{\left(\frac{\omega_Q}{\omega_1}\right)^2-\frac{\omega_Q}{\omega_1}+1} \tag{S1c}$$

S2. Comparison of spin-lattice relaxation curves calculated using the exact expression in eq S1 and the approximate expression in eq 16

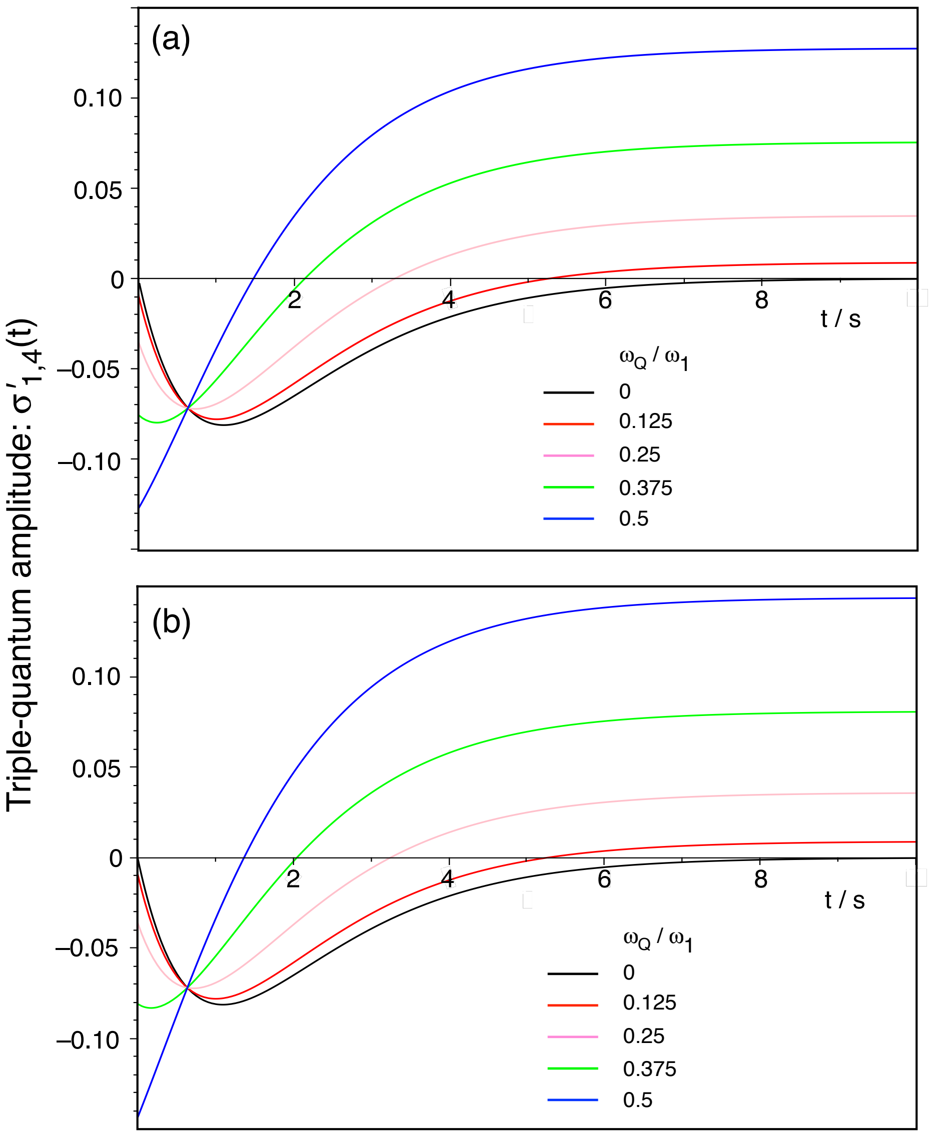

Figure S2. Plots of (a) $\sigma'_{1,4}(t)$ from eq S1 (a theoretical measure of the amplitude of I = 3/2 triple-quantum coherence excited by a 90° pulse) and (b) the approximation $\sigma'^{(approx)}_{1,4}(t)$ from eq 16 as a function of the spin-lattice relaxation interval t in a triple-quantum filtered inversion-recovery experiment ($\alpha = -1$). The plot in (a) is a repeat of that in Fig. 2a (so with $C_Q = 30$ kHz, $\eta = 0$, $\omega_0/(2\pi) = 155$ MHz and $\omega_0\tau_c = 0.3$) while the relaxation curves in (b) can be seen to only differ significantly from those in (a) when $\omega_Q/\omega_1 = 0.5$. This demonstrates the approximate validity of eq 16 when $\omega_Q/\omega_1 < 0.5$.

S3. Crystallographic and powder X-ray data for the prepared sample of Li_2OHCl

Figure S3a. Powder X-ray diffraction pattern for Li_2OHCl at 25 °C (Mo K_α: λ = 0.7107 Å):

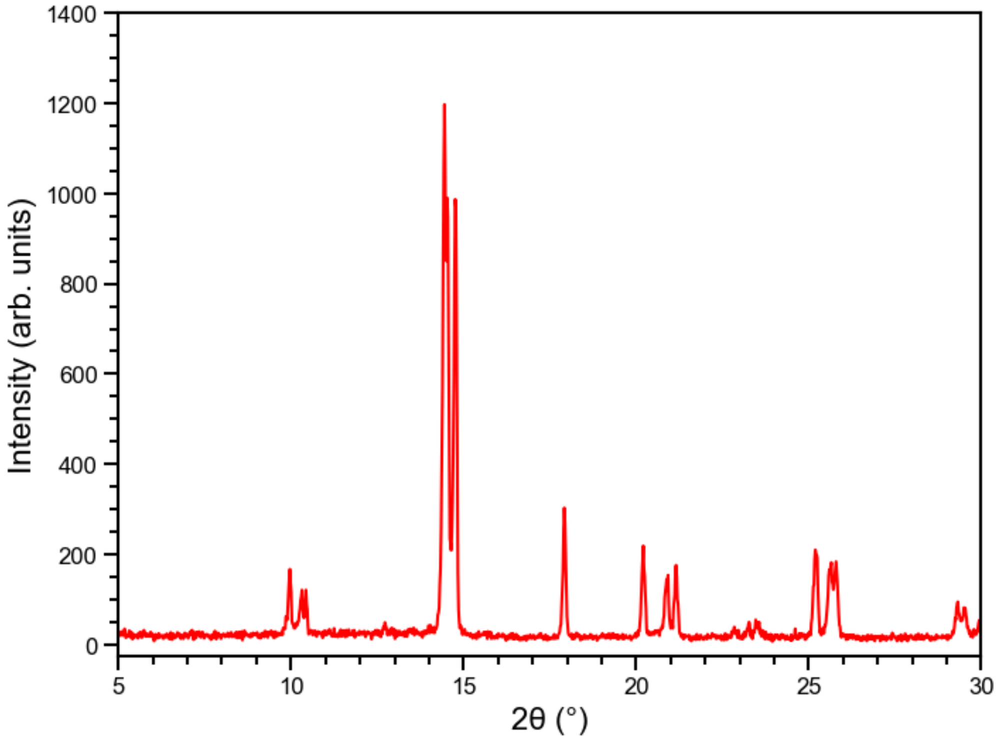

Figure S3b. Powder X-ray diffraction pattern for Li_2OHCl at 50 °C (Mo K_α: λ = 0.7107 Å):

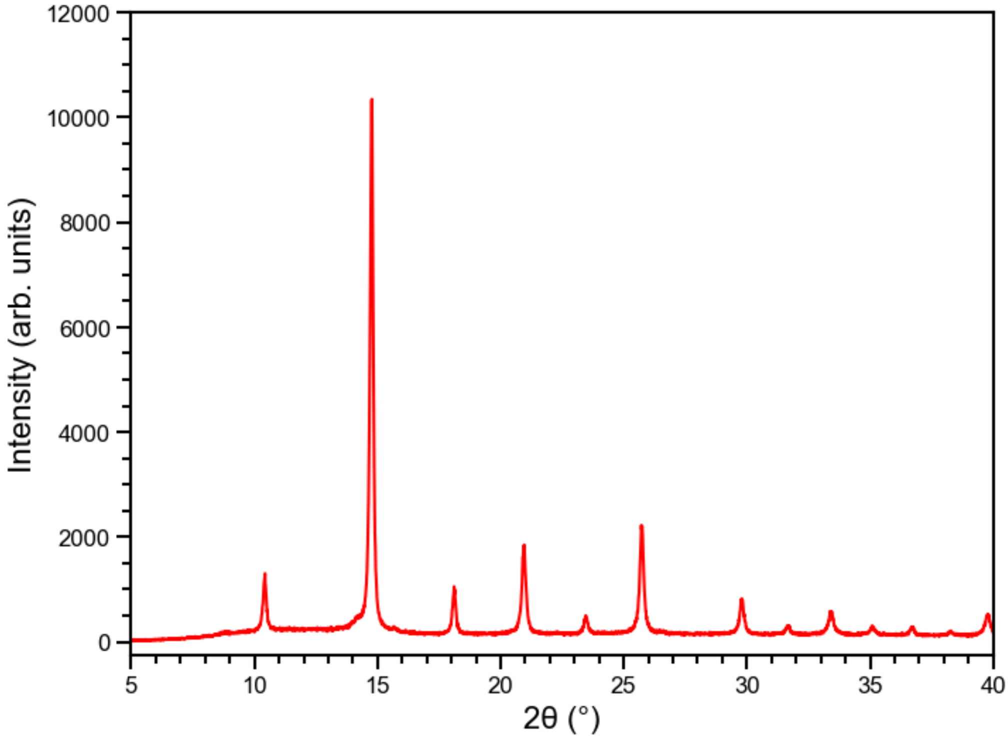

Figure S3c. Overlay of powder X-ray diffraction patterns:

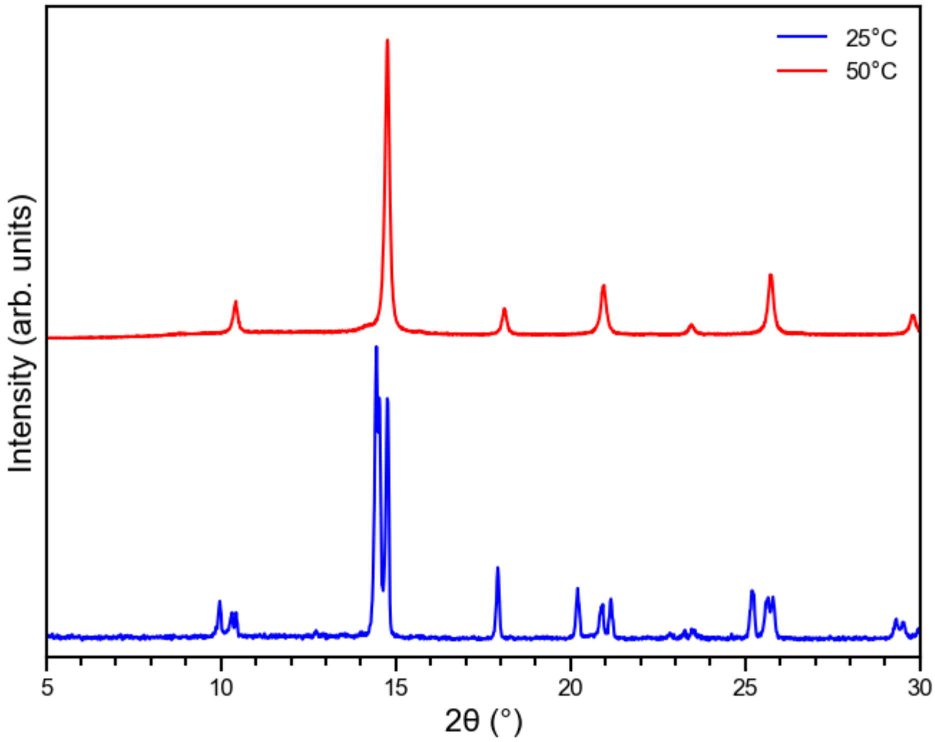

Figure S3d. Rietveld refinement of the powder X-ray diffraction data for Li_2OHCl at 25 °C:

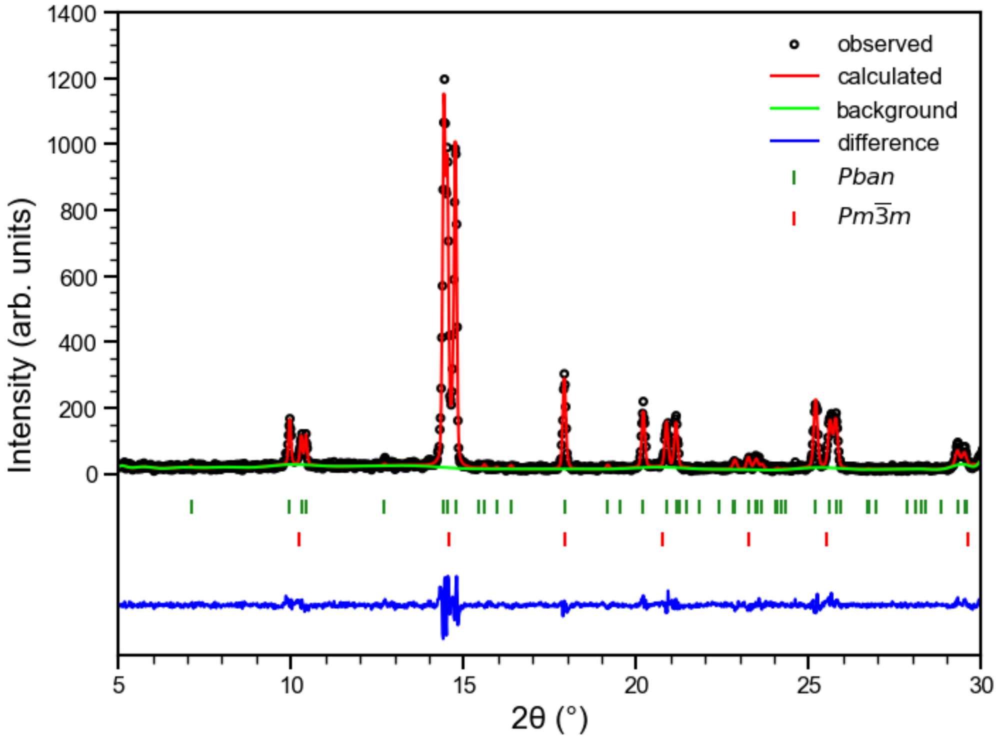

Table S3a. Refinement parameters for Li_2OHCl at 25 °C:

Phase	*a* (Å)	*b* (Å)	*c* (Å)	α (°)	β (°)	γ (°)	V (Å^3)	Weight Fraction (%)
Pban	7.7534(9)	8.0054(9)	3.8260(4)		90		237.47(8)	97.72(3)
Pm$\bar{3}$*m*		3.8691(7)			90		57.9(3)	2.28(3)

R_{wp} = 18.56%
R_p = 13.94%
χ^2 = 1.52
GOF = 1.23

Figure S3e. Rietveld refinement of the powder X-ray diffraction data for Li_2OHCl at 50 °C:

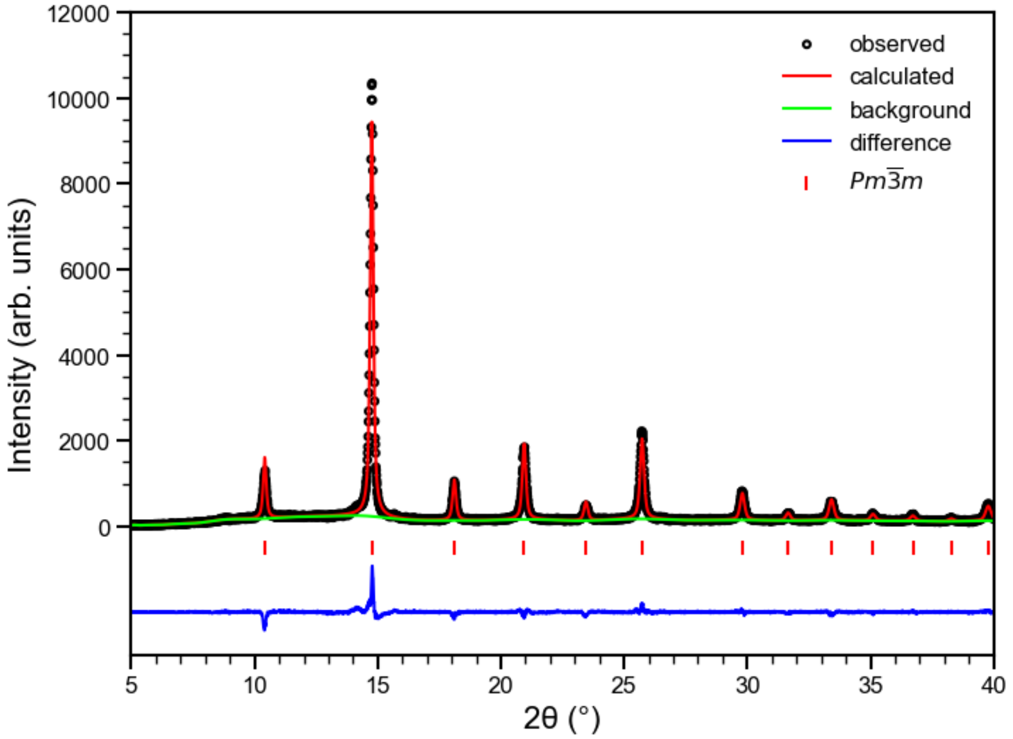

Table S3b. Refinement parameters for Li_2OHCl at 50°C:

Phase	*a* (Å)	*b* (Å)	*c* (Å)	α (°)	β (°)	γ (°)	V (Å^3)
Pm$\bar{3}$*m*	3.8974(2)			90			59.20(8)

R_{wp} = 9.48%
R_p = 7.45%
χ^2 = 2.30
GOF = 1.52

Table S3c. Crystallographic details for the Li_2OHCl phases at 25 °C:

Pban	Species	x	y	z	Occupancy
	Li1	−0.07000	0.04000	−0.41200	1/3
	Li2	0.32400	0.40000	−0.89700	1/3
	Li3	0.03400	−0.22900	−0.10800	1/3
	O1	0.00000	0.00000	0.00000	1.000
	Cl1	0.25000	0.25000	0.50000	1.000
	Cl2	0.75000	0.25000	0.50000	1.000

$Pm\bar{3}m$	Species	x	y	z	Occupancy
	Li1	0.50000	0.00000	0.00000	2/3
	O1	0.00000	0.00000	0.00000	1.000
	Cl1	0.50000	0.50000	0.50000	1.000

Table S3d. Crystallographic details for the cubic Li_2OHCl phase at 50 °C:

$Pm\bar{3}m$	Species	x	y	z	Occupancy
	Li1	0.50000	0.00000	0.00000	2/3
	O1	0.00000	0.00000	0.00000	1.000
	Cl1	0.50000	0.50000	0.50000	1.000

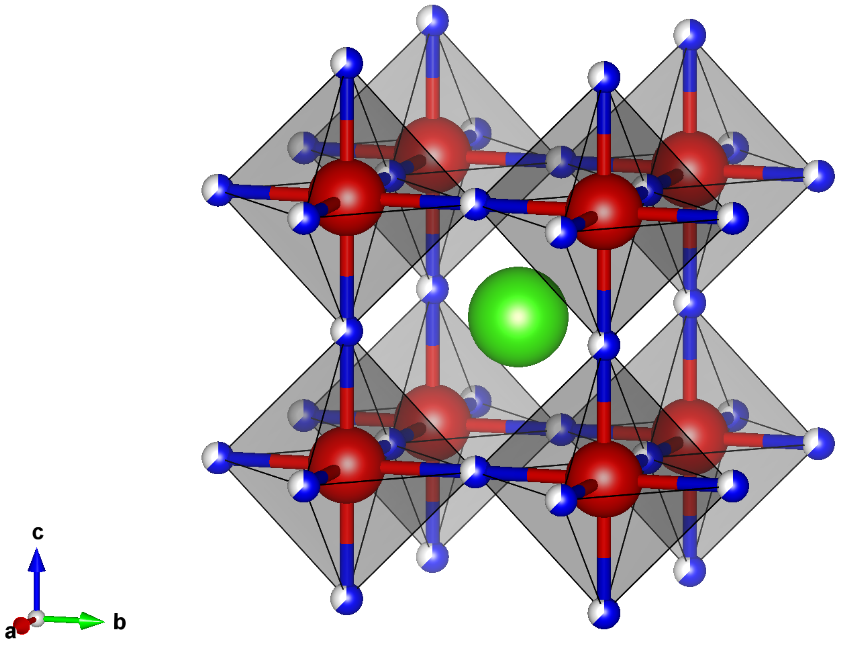

Figure S3f. Crystal structure of Li_2OHCl (cubic, $Pm\bar{3}m$). Colour scheme: blue = Li, red = O and green = Cl.
